# ALCAM Regulates Motility, Invasiveness, and Adherens Junction Formation in Uveal Melanoma Cells

**DOI:** 10.1371/journal.pone.0039330

**Published:** 2012-06-26

**Authors:** Karry M. Jannie, Christopher S. Stipp, Joshua A. Weiner

**Affiliations:** Department of Biology, The University of Iowa, Iowa City, Iowa, United States of America; University of Michigan, United States of America

## Abstract

ALCAM, a member of the immunoglobulin superfamily, has been implicated in numerous developmental events and has been repeatedly identified as a marker for cancer metastasis. Previous studies addressing ALCAM’s role in cancer have, however, yielded conflicting results. Depending on the tumor cell type, ALCAM expression has been reported to be both positively and negatively correlated with cancer progression and metastasis in the literature. To better understand how ALCAM might regulate cancer cell behavior, we utilized a panel of defined uveal melanoma cell lines with high or low ALCAM levels, and directly tested the effects of manipulating these levels on cell motility, invasiveness, and adhesion using multiple assays. ALCAM expression was stably silenced by shRNA knockdown in a high-ALCAM cell line (MUM-2B); the resulting cells displayed reduced motility in gap-closure assays and a reduction in invasiveness as measured by a transwell migration assay. Immunostaining revealed that the silenced cells were defective in the formation of adherens junctions, at which ALCAM colocalizes with N-cadherin and ß-catenin in native cells. Additionally, we stably overexpressed ALCAM in a low-ALCAM cell line (MUM-2C); intriguingly, these cells did not exhibit any increase in motility or invasiveness, indicating that ALCAM is necessary but not sufficient to promote metastasis-associated cell behaviors. In these ALCAM-overexpressing cells, however, recruitment of ß-catenin and N-cadherin to adherens junctions was enhanced. These data confirm a previously suggested role for ALCAM in the regulation of adherens junctions, and also suggest a mechanism by which ALCAM might differentially enhance or decrease invasiveness, depending on the type of cadherin adhesion complexes present in tissues surrounding the primary tumor, and on the cadherin status of the tumor cells themselves.

## Introduction

The immunoglobulin superfamily (IgSF), a class of proteins with 765 putative members in humans [1] represents one of the most ancient and diverse families of cell adhesion proteins. Not surprisingly, IgSF members are key players in numerous developmental and pathological processes [2]–[5]. Activated Leukocyte Cell Adhesion Molecule (ALCAM; also reported as CD166, DM-GRASP, neurolin, and BEN), an IgSF member, has been implicated in the regulation of many developmental events, including hematopoiesis [6]–[8], osteogenesis [9], T cell activation [10]–[12], and neurite outgrowth, fasciculation, and targeting [13]–[21]. Some of these studies suggested roles for ALCAM based on its expression pattern alone, while others utilized a variety of in vitro assays to identify ALCAM functions. To assess *in vivo* roles, we generated the first null mutation of ALCAM in any model organism by targeting the *Alcam* locus in mice [22]. ALCAM-null mice are viable and fertile, suggesting that ALCAM’s functions *in vivo* may not be as broad as assumed from these earlier studies, although a high degree of functional redundancy among IgSF members is also likely.

Nevertheless, we found that ALCAM-null mice do display several nervous system defects predicted by previous studies, including disrupted fasciculation of both motor and retinal ganglion cell axons [22], and mistargeting of retinal ganglion cell axons within the superior colliculus [23]. In addition, ALCAM-null mice on a mixed C57BL/6-129 background exhibit retinal dysplasias, including disrupted organization of the outer nuclear layer photoreceptor neurons and invagination of the adjacent retinal pigment epithelium (RPE) and choroid (choriocapillaris) [22]; these dysplasias are greatly reduced on a congenic C57BL/6 background, however (data not shown). This last phenotype was initially puzzling, since expression of ALCAM in the retina is restricted to retinal ganglion cells and a subset of inner nuclear layer amacrine cells, with no expression detectable in photoreceptor neurons or the RPE. We found, however, high levels of ALCAM expression in melanocytes and stromal cells of the choroid [22], a pigmented tissue that nourishes the RPE and photoreceptor cells and provides much of the blood supply to the eye (for review see ref. [24]). This previously undocumented expression, as well as the fact that choroidal melanocytes were found within ectopic retinal folds [22] suggests that in the absence of ALCAM, the structure and/or function of melanocytes in the uvea, which includes the choroid, iris, and ciliary body, might be disrupted.

We found this phenotype to be particularly interesting in light of dozens of reports identifying ALCAM as a potential regulator of tumor cell behavior. Indeed, ALCAM has been implicated in the progression and metastasis of cutaneous melanoma [25], prostate carcinoma [26], [27], breast cancer [28]–[30], colorectal carcinoma [31], bladder cancer [32], and esophageal squamous cell cancer [33], among others (for review see ref. [5]). Although ALCAM has been implicated in these numerous pathological states, it is as yet unclear how ALCAM contributes to metastasis. Existing reports are paradoxical, with ALCAM gene expression being highly upregulated in some cancers [25], [31], [34] and greatly downregulated in others [28], [35], [36]. Unfortunately, these data are necessarily correlative in nature; therefore, an understanding of the contribution of ALCAM to cancer progression and, indeed, normal cell motility and adhesion, has been hampered by a lack of studies aimed at directly manipulating ALCAM levels within particular cell lines and determining the outcome of this manipulation.

Here, we sought to address this by utilizing a number of defined uveal melanoma cell lines with high or low ALCAM levels, and testing the effects of manipulating these levels on cell motility, invasiveness, and adhesion using multiple measures. Uveal melanoma, the most common form of primary intraocular cancer, is often derived from the choroid, is highly metastatic, and results in death in 50% of patients [37]. Previous microarray analysis of two uveal melanoma cell lines identified ALCAM as one of the genes most upregulated in invasive cells (line MUM-2B) compared to non-invasive cells (line MUM-2C) [38]. We find that, across several uveal melanoma cell lines, ALCAM expression positively correlates with cell motility. Silencing of ALCAM using targeted shRNAs in MUM-2B results in both impaired cell motility and reduced invasive capacity in an *in vitro* assay, consistent with an observed reduction in matrix metalloproteinase activation. Conversely, forced expression of ALCAM in the normally ALCAM-negative line MUM-2C did not increase cell motility or invasiveness, demonstrating that ALCAM is necessary but not sufficient for this cell behavior. Interestingly, we also find an effect of ALCAM on cadherin-based adherens junctions. In MUM-2C cells, forced expression of ALCAM results in increased recruitment of neural (N)-cadherin and ß-catenin to cell-cell contacts. Conversely, silencing of ALCAM expression in MUM-2B disrupts the formation of adherens junctions. These data represent the first description of ALCAM function in uveal melanoma cells, indicate cooperation between ALCAM and cadherins in mediating cell adhesion, and suggest that ALCAM’s ultimate effect on metastasis might depend on the cadherin status of surrounding tissues in conjunction with the cadherin status of the tumor cells.

## Methods

### Reagents, Cell Culture Media, Antibodies

DMEM, RPMI-1640, Anti-GFP (rabbit), trypsin-EDTA solution, HEPES, EHS laminin and all fluorescent-tagged secondary antibodies were from Invitrogen (Carlsbad, CA, USA). Anti-N-cadherin and anti- ß-catenin mouse monoclonal antibodies were purchased from Becton Dickinson (Franklin Lakes, NJ, USA). Anti-MMP-2 rabbit polyclonal antibody was purchased from Santa Cruz Biotechnology (Santa Cruz, CA, USA). Sodium orthovanadate, anti-pan-cadherin, anti- ß-tubulin, and poly-L-lysine (0.01%) were purchased from Sigma-Aldrich (St. Louis, MO, USA). Anti-GFP (mouse) and protease inhibitor cocktail EDTA-free mini tablets were from Roche (USA). Supersignal West Pico reagents, BCA assay reagents, and HRP-conjugated anti-mouse and anti-rabbit secondary antibodies were supplied by Thermo Scientific (Rockford, IL, USA). HRP-conjugated anti-goat secondary antibody was purchased from Jackson ImmunoResearch (West Grove, PA, USA). Fetal Bovine Serum (FBS) was purchased from Atlanta Biologicals (Lawrenceville, GA, USA). Mouse monoclonal anti-ALCAM 3A6 antibody was purchased from Millipore (Billerica, MA, USA). Goat anti-ALCAM AF656 was purchased from R&D Systems (Minneapolis, MN, USA). Anti-ALCAM HB-2 antibody was the kind gift of Solomon Ofori-Acquah (Emory University School of Medicine, GA, USA). Puromycin and G418 were supplied by InvivoGen (San Diego, CA, USA) and Research Products International Corp. (Mt. Prospect, IL, USA), respectively.

### Cell Culture Conditions

Cell lines were grown as follows: human uveal melanoma cell lines OCM-1A, MUM-2B, MUM-2C, C918, M619, sh5, sh6, sh5rxd, and 2C-ALC were maintained in RPMI-1640 supplemented with 10% FBS; selection was maintained in sh5 and sh6 with 1 µg/ml puromycin; in sh5rxd with 1 µg/ml puromycin and 250 µg/ml G418; in 2C-ALC with 250 µg/ml G418. HEK cells and the retroviral packaging cell line GP2-293 was maintained in DMEM supplemented with 10% FBS. All cell lines were maintained at 37 degrees Celsius in 5% carbon dioxide. The MUM-2B and MUM-2C lines were the kind gift of Dr. Elizabeth Seftor (Children’s Memorial Research Center, Chicago, IL, USA); the OCM-1A, C918, and M619 lines were the kind gift of Dr. Karla Daniels (University of Iowa, Iowa City, IA, USA).

### Immunohistochemistry

Cells were grown in 24-well dishes on glass coverslips coated with poly-L-lysine (0.01%) and laminin (10 µg/ml). Cells were grown on coverslips until at the desired confluency, then fixed for 15 minutes in 4% paraformaldehyde, rinsed with 1×PBS, and blocked in standard blocking solution (2.5% BSA, 0.1% Triton X-100, 0.02% sodium azide) for 1 hour. Primary antibodies were diluted in blocking solution and incubated on coverslips overnight at 4 degrees Celsius. Coverslips were rinsed in 1×PBS and secondary antibody diluted in 1×PBS was added for 1 hour at room temperature. Coverslips were rinsed in 1×PBS containing DAPI (4′,6-diamidino-2-phenylindole) to stain nuclei, then rinsed once with water and set on filter paper to dry. After drying, coverslips were mounted on glass slides using GelMount (Biomeda Corporation, Foster City, CA, USA). ß-catenin-positive regions between two cells were scored as strong, weak, or non-existent as shown below in the text and figures. Contacting cells in a minimum of three independent fields were scored.

### Plasmids and Vectors

The sh5 and sh6 cell lines were created using viral transduction of shRNA constructs in the pSIREN-RetroQ vector. Hairpin sequences were cloned into the vector between 5′ BamHI and 3′ EcoRI sites.

The sequence of the sh5 insert is: 5′-GAT CCG TAT GTC TGC GAA ACT GCT CTG TTC AAG AGA CAG AGC AGT TTC GCA GAC ATA TTT TTT CTA GAG-3′.

The sequence of sh6 is: 5′-GAT CCG TCA AGC AAC CAT CTA AAC CTG TTC AAG AGA CAG GTT TAG ATG GTT GCT TGA TTT TTT CTA GAG-3′. The shRNA-containing plasmids were cotransfected into GP2-293 cells along with pCMV-VSV-G to produce virus particles.

The 2C-ALC cell line was created by viral transduction of pWD201 into MUM-2C cells. The plasmid was previously described in [39].

To create the sh5rxd cell line, sh5 cells were transduced a second time with the construct pWD-ALCAM-GFP-mut3. This plasmid was created by first cloning full-length ALCAM from pWD201 into pEGFPN1 at SalI and AgeI sites to create pEGFPN1-ALCAM. To avoid shRNA knockdown of the rescue plasmid, three silent mutations were introduced into this plasmid by use of the Stratagene QuikChange kit, using the following primers: Forward- TGC TGG AAA CTA TGT TTG TGA GAC TG; Reverse – CTC CTG CAG AGC AGT CTC ACA AAC AT. The resulting plasmid, pEGFPN1-ALCAM-mut3 was subjected to PCR using primers containing BamHI and EcoRI ends to yield a full-length ALCAM fragment tagged at the C-terminus with GFP. This fragment was cloned into the pLXIN vector to yield pLXIN-ALCAM-GFP-mut3.

pCMV-VSV-G, encoding a viral envelope protein was the kind gift of Chris Stipp (University of Iowa, Iowa City, IA, USA).

The HEK-sh0 and HEK-sh2 cell lines were created by transient transfection of HEK cells using Lipofectamine 2000 (Invitrogen, Carlsbad, CA, USA) with either a negative control (sh0) construct in pGeneClip-hMGFP, or an shRNA against ALCAM (sh2) in pGeneClip-hMGFP (SABiosciences, Valencia, CA, USA). The sequence of the sh0 hairpin is: 5′ – TCT CGG AAT CTC ATT CGA TGC ATA CCT TCC TGT CAG TAT GCA TCG AAT GAG ATT CCC T –3′. The sequence of the sh2 hairpin is: 5′ – TCT CGT CAG GAT GCT GGA AAC TAT GTC TTC CTG TCA ACA TAG TTT CCA GCA TCC TGA CT –3′.

### Retroviral Transduction

The stable cell lines sh5, sh6, 2C-ALC, and sh5rxd were created by retroviral transduction. The appropriate constructs were cotransfected, along with pCMV-VSV-G, into the GP2-293 packaging cell line. Conditioned media was harvested from the GP2-293 cells, filtered, supplemented with 4 µg/ml polybrene, and added to the appropriate parental cell line. The following day, the media was removed from the parental cell line, and conditioned media from the GP2-293 cells was added again. This process was repeated a total of three times. After the final day of incubation in conditioned media, the parental cells were grown for 24 hours in standard growth medium, and then appropriate selection agents were added.

### Gap Closure Assay/time-lapse Imaging

Confluent cultures were grown in 6 cm dishes. A 10 µl pipet tip was used to inscribe a “wound gap” in the confluent layer of cells. Cells were washed twice with 1×PBS, then RPMI-1640 media containing 10% FBS and 25 mM HEPES was added. The dishes were covered and placed on a custom stage heater that maintained media temperature at ∼37 degrees Celsius. To analyze cell motility, phase contrast time-lapse microscopy was done using a QICAM camera (Q Imaging, Surrey, British Columbia, Canada) and 10× objective on a Leica DM-IRB microscope. Images were collected every 10 minutes for 8 hours, or until cells closed the wound gap. Analysis of cell migration was conducted by using QImagePro (Q Imaging, Surrey, British Columbia, Canada). The initial width of the gap was measured at three different places along the length of the wound, and the time until the cells closed the gap was recorded for each. The three speeds were calculated and averaged together to yield a value for that trial. Each reported speed represents at least three independent trials.

### RNA, cDNA, and RT-PCR

RNA was extracted from 10 cm dishes of confluent cells using Trizol™ reagent (Invitrogen, Carlsbad, CA, USA). RNA was subsequently reverse transcribed into cDNA using the ProtoScript FirstStrand cDNA kit (NEB, Ipswich, MA, USA). cDNA was then subjected to PCR (30–35 cycles) using primers that spanned an intron of either ALCAM, MMP-2, or GAPDH.

### Preparation of Cell Lysates

Cells were grown until confluent in 10 cm dishes, growth media was aspirated, and cells were washed twice with 1×PBS. To a 10 cm dish, 1 ml of Mild Lysis Buffer (1% NP-40, 100 mM Tris-HCl pH7.4, 300 mM NaCl, 10 mM NaF, 0.5 mM sodium orthovanadate, 1 protease inhibitor cocktail EDTA-free mini tablet per 10 ml) was added. Cells were incubated for 10 minutes on ice, then scraped into an eppendorf tube. The lysate was spun at 4 degrees Celsius in a table top microcentrifuge for 10 minutes at 16,000 g to pellet cell debris. The protein concentration of the resulting supernatant was quantified using a BCA assay.

### Western Blots

For all western blots, 20 micrograms of total protein was loaded per sample. For all western blots except MMP-2, lysates were run on 9% acrylamide running gels with a 4% acrylamide stacking gel. Lysates blotted for MMP-2 were run on precast 4–20% gradient gels. Gels were run at 185 volts constant. Protein was then transferred to 0.45 µm nitrocellulose using a ThermoFisher electrotransfer apparatus at 0.4 amps constant. After transfer, the nitrocellulose membrane was blocked for 1 hour at room temperature in 5% dry milk in 0.05% Tween20-TBS (for rabbit and mouse primary antibodies) or in 0.05% Tween20-TBS (for goat primary antibodies). Primary antibodies were diluted in blocking solution and incubated overnight at 4 degrees Celsius. The blots were washed 3 times in 0.05% Tween20-TBS, and secondary antibodies were diluted in 0.05% Tween20-TBS and incubated with the blot for 1 hour at room temperature. The blots were washed with 0.05% Tween20-TBS three times, then developed using West Pico reagents (ThermoFisher Scientific, Rockford, IL, USA).

### Gelatinase Assay

Cells were grown to confluency in a 10 cm dish. Growth medium was removed and replaced with 5 ml of serum-free medium for 36 hours. The media were harvested, filtered through a 0.45 micron filter to remove cells and debris, and mixed 1∶1 with non-reducing sample buffer. Samples were treated as in [40], and 10 µl loaded onto an SDS-PAGE gel that included 0.1% gelatin.

### Invasion Assay

The CytoSelect™ 24-well Invasion Assay, Basement Membrane, Colorimetric format (Cell Biolabs, Inc., San Diego, CA, USA) was used according to manufacturer’s protocol. Briefly, 300 µl of a 0.5×10^6^ cells per milliliter solution were plated in serum-free RPMI-1640 in the upper chamber of an insert. The bottom portion of the well was filled with 500 µl of RPMI-1640 containing 10% FBS and penicillin-streptomycin. Cells were incubated for 8 hours at 37 degrees Celsius, 5% carbon dioxide. After incubation, inserts containing invasive cells were removed and stained with the provided cell stain solution. Stained cells in three non-overlapping fields centered at the highest cell density were counted at 10× magnification on an inverted microscope.

### Survival Assay

Cells were trypsinized, counted on a hemocytometer, and 120,000 cells were plated in triplicate in single wells of a 24-well plate. The cells were incubated for 8 hours at 37 degrees Celsius and 5% carbon dioxide, then trypsinized and counted on a hemocytometer.

### Flow Cytometry

To maintain a homogeneous population of ALCAM-expressing or ALCAM-silenced cells, the sh5, 2C-ALC, and sh5rxd cell lines were flow-sorted using a FACSDiva (Becton Dickinson, Franklin Lakes, NJ, USA). Both sh5 and 2C-ALC were sorted using the anti-ALCAM 3A6 antibody (Millipore, Billerica, MA, USA) and phycoerythrin anti-mouse secondary antibody. For sh5, low-ALCAM cells were kept and high ALCAM cells were discarded. For 2C-ALC, ALCAM-positive cells were kept, and ALCAM-negative cells were discarded. The sh5rxd cell line was sorted using the cytoplasmic GFP tag on the full-length ALCAM construct they were transduced with; GFP-positive cells were kept and GFP-negative cells were discarded.

## Results

### ALCAM Expression Correlates with Cell Motility in Uveal Melanoma Cell Lines

To begin to address the role of ALCAM in tumor cell behavior, we assembled a panel of five uveal melanoma cell line stocks: OCM-1A, MUM-2B, MUM-2C, C918, and M619. All cell lines had been previously characterized by cell phenotype, invasive potential, and vasculogenic mimicry [38]–[42]. Two of the lines, OCM-1A and MUM-2C, are poorly invasive and resemble normal uveal melanocytes, while the remaining three cell lines were characterized as highly invasive, based on the ability to invade a collagenous matrix-coated polycarbonate membrane. MUM-2B and MUM-2C were initially reported to be isolated from the same metastasis from a primary uveal melanoma, and found to be phenotypically divergent: MUM-2B is epithelioid, while MUM-2C is spindle-shaped [38]. Microarray analysis of these two lines revealed that ALCAM was one of the most up-regulated genes (8.3-fold) in highly invasive MUM-2B cells versus poorly invasive MUM-2C cells [38]. Subsequent analysis of short tandem repeats in the genomic DNA of these uveal melanoma lines indicates that MUM-2B and MUM-2C are, in fact, unlikely to have derived from the same metastasis [43]. Folberg and colleagues (2008) additionally present evidence that OCM-1A and MUM-2C share the same origin, as do MUM-2B, M619, and C918; our data below are consistent with this. Therefore, while we initially examined all five cell lines, most of our work has focused on MUM-2B and MUM-2C as exemplars.

**Figure 1:**
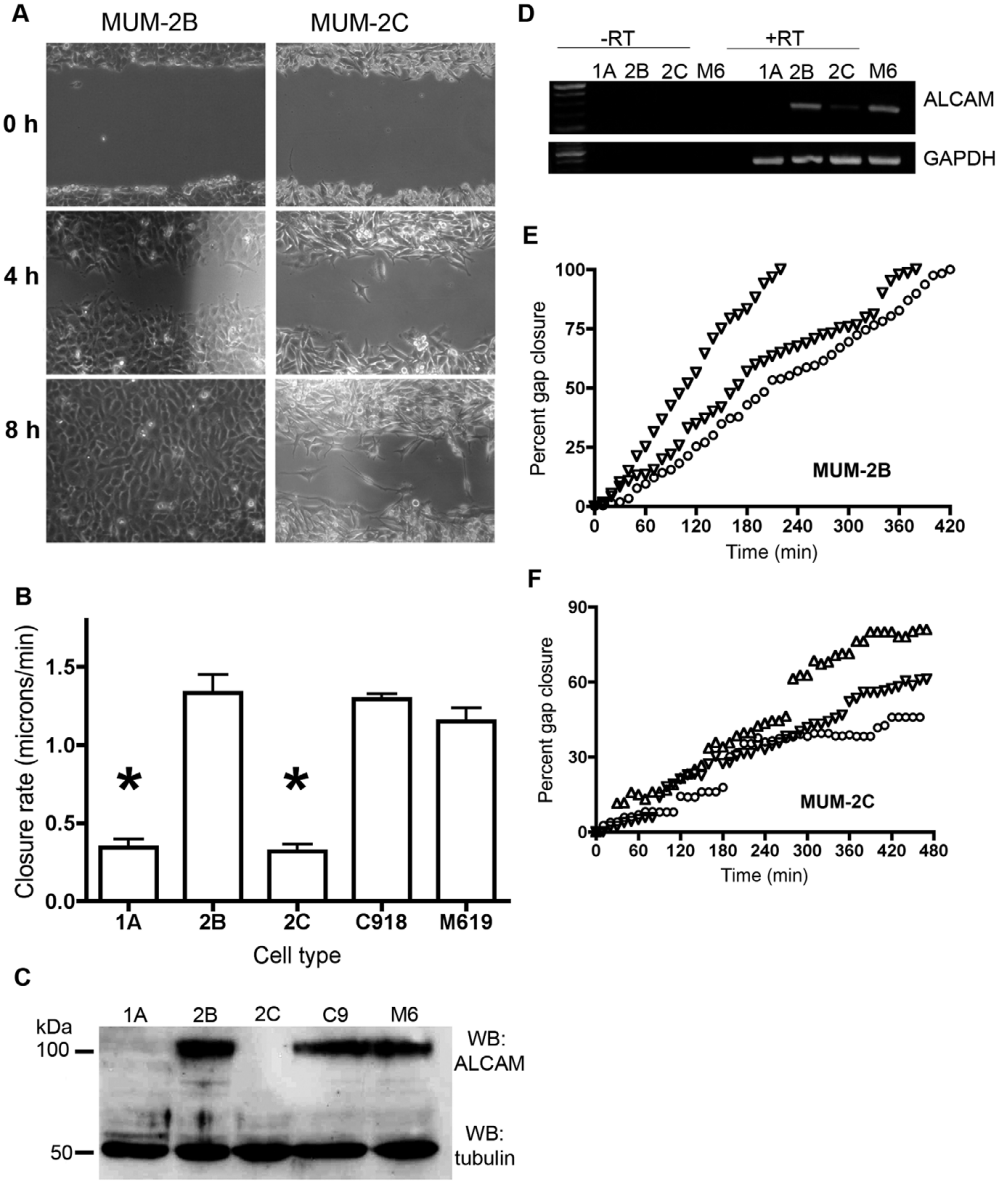
Uveal melanoma cell lines display different speeds of migration that correlate with ALCAM expression. Time-lapse imaging of a wound created with a pipet tip in a layer of confluent cells (A) reveals the difference in wound closure in MUM-2B vs. MUM-2C. The speed of 5 uveal melanoma cell lines (minimum 3 trials each) is quantified (B). Both OCM-1A and MUM-2C move at less than 0.5 microns per minute; MUM-2B, C918, and M619 move at approximately 1.2 microns per minute. The speed of OCM-1A and MUM-2C are significantly different when compared to MUM-2B (t-test; 1A vs. 2B, p = 0.0008; 2C vs. 2B, p<0.0001). ALCAM protein expression as assayed by western blot shows that the cell lines fall into two groups – those with detectable ALCAM, and those without detectable protein expression (C). Tubulin is shown as a loading control. RT-PCR analysis of 4 of the 5 cell lines reveals that ALCAM mRNA expression mirrors protein expression (D). GAPDH is shown as a positive control. Percent gap closure was tracked for MUM-2B (E) and MUM-2C (F), and revealed that these two representative cell lines differ in their modes of migration. While the tracking of the wound front is fairly linear in MUM-2B, the cell front of MUM-2C advances in a stop-and-start manner, due to loosely associated single cells progressing into the gap. Error bars are mean ± S.E.M.

We first utilized a gap-closure assay as one measure of the motility of each cell line. Freshly confluent monolayers were inscribed with a gap using a micropipet tip, and movement of cells back into the gap was monitored by time-lapse imaging. MUM-2B cells moved more quickly to close the gap than did MUM-2C cells, and had completely closed the gap by 8 hours (Fig. 1A). Time-lapse analysis showed that MUM-2B cells appeared to move as a cohesive sheet across the empty space of the gap (Fig. 1A). Contrastingly, MUM-2C cells failed to completely close the gap by 8 hours, and, unlike MUM-2B, individual cells could be seen breaking away from the cell front and moving across the gap space individually (Fig. 1A). Gap closure analysis of the remaining cell lines revealed that M619 and C918 were fast-moving like MUM-2B, while OCM-1A was slow-moving like MUM-2C. The average speed of each cell line was calculated from three independent gap closure trials (Fig. 1B). MUM-2B, C918, and M619 all moved at speeds 2–3 fold greater than OCM-1A and MUM-2C (Fig. 1B).

Next, we assayed ALCAM expression in the five cell lines by both western blot (Fig. 1C) and RT-PCR (Fig. 1D). ALCAM protein expression was undetectable in OCM-1A and MUM-2C; in contrast, it was similarly high in MUM-2B, C918, and M619 (Fig. 1C). ALCAM can be shed from the membrane via the action of ADAM-10 and ADAM-17 metalloproteinases [44], [45]; therefore we asked whether the lack of ALCAM protein in MUM-2C and OCM-1A indicated a true lack of gene expression or simply accelerated shedding and/or degradation. RT-PCR analysis of cDNA demonstrated that few, if any, ALCAM transcripts are present in these two cell lines, supporting the former possibility (Fig. 1D). Finally, we analyzed the mode of migration of each cell line, by plotting the cumulative percent of the initial gap closed versus time: Three separate trials of the gap-closure assay in MUM-2B (Fig. 1E) and MUM-2C (Fig. 1F) are shown. As noted above, MUM-2B appeared to move as a cohesive sheet to close the gap, and this linear mode of movement is reflected in the plots (Fig. 1E). In contrast, MUM-2C did not seem to move as a cohesive sheet, but instead, individual cells extended filopodia and the cell front moved discontinuously to close the gap. This is apparent in Figure 1F, where discrete “jumps” on the Y-axis demonstrate this stop-and-start movement. Together, these data demonstrate that ALCAM expression positively correlates with cell motility in uveal melanoma cell lines.

### Establishment of ALCAM-silenced MUM-2B Cell Lines by shRNA Knockdown

**Figure 2:**
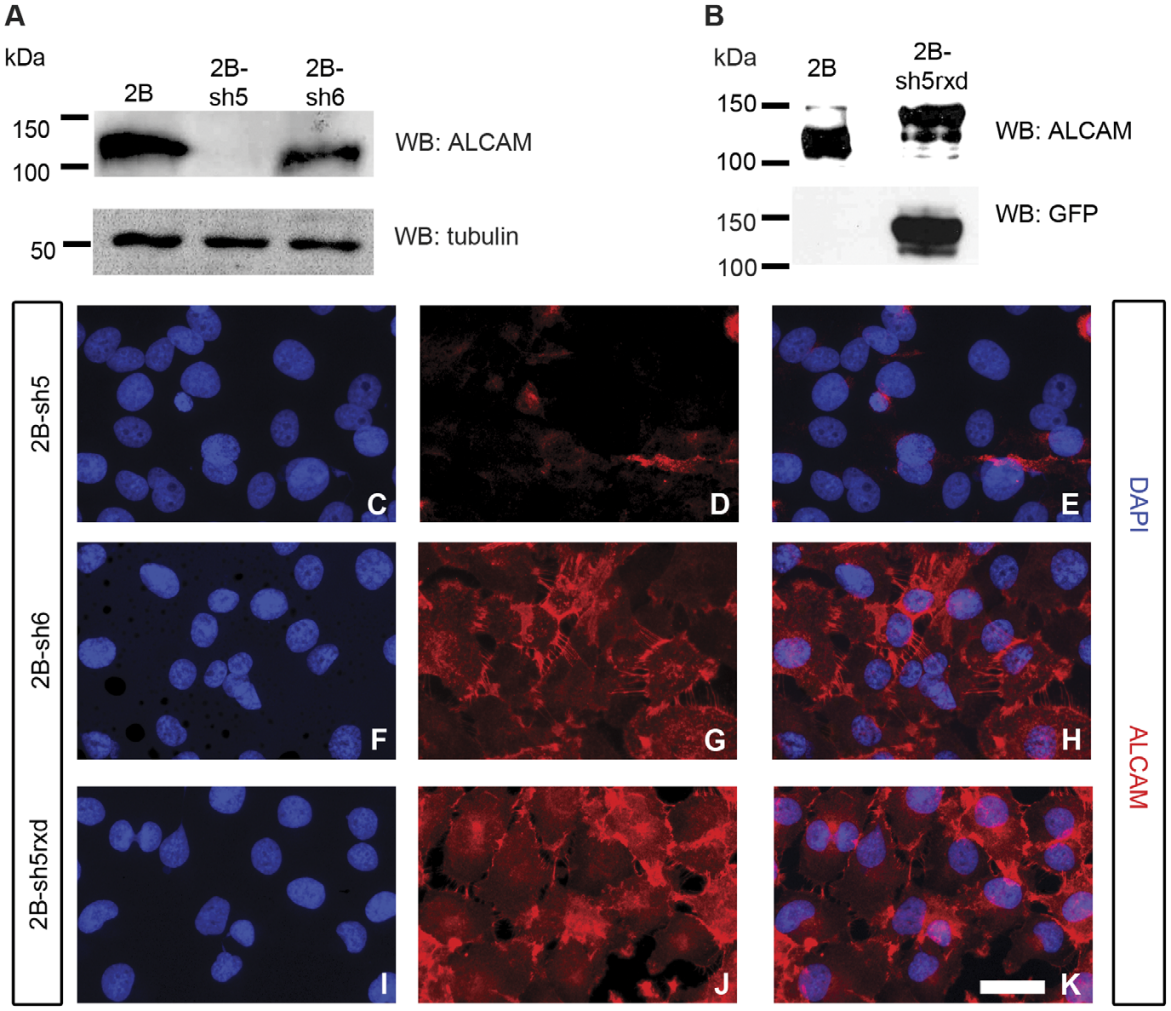
ALCAM expression is silenced in MUM-2B cells via shRNA, and rescued by re-expression of ALCAM-GFP. Western blots of MUM-2B, ALCAM-silenced sh5, control sh6, and negative control MUM-2C reveal that ALCAM protein expression is nearly completely silenced in sh5, but not in sh6 (A). Tubulin is shown as a loading control. ALCAM expression in the rescue cell line (sh5rxd, containing an ALCAM-GFP construct with point mutations to evade knockdown) is similar to MUM-2B, as shown by western blot using an anti-ALCAM antibody (B; note that the GFP tag adds ∼27 kDa to the size of the protein; reprobing the blot with anti-GFP antibody detects only the rescue band, as expected). Panel (C) shows DAPI-stained nuclei of the sh5 cell line; immunostaining of sh5 confirms nearly complete knockdown of ALCAM protein expression (D, E). Panel (F) shows DAPI-stained sh6 nuclei; ALCAM expression in this cell line is robust, and localized to points of cell contact (G, H). Panel (I) shows DAPI-stained nuclei of the sh5rxd rescue cell line; ALCAM expression and localization in the rescue cells (J, K) is comparable to the sh6 cell line. Scale bar in (K) is 25 microns.

To determine whether ALCAM regulates uveal melanoma cell behavior, we began by knocking down ALCAM levels in MUM-2B cells, which normally express high levels of ALCAM. This was accomplished via transduction with retroviral constructs encoding shRNAs targeted against the *Alcam* transcript. We tested a total of 6 different shRNA sequences, and focus here on two such constructs, termed sh5 and sh6. Initial immunostaining of MUM-2B cells infected with virus particles showed that many, though not all, sh5-expressing cells completely lost detectable ALCAM, while sh6 expression failed to silence ALCAM expression detectably (data not shown). To isolate a purified population of silenced MUM-2B cells, we performed FACS sorting using an antibody against the ALCAM ectodomain, keeping only the population of sh5-expressing cells that lacked detectable ALCAM. These cells, termed sh5 cells, were utilized for experiments, using sh6-expressing cells as well as parental MUM-2B cells as control groups (Fig 2A, C–H).

To confirm that any phenotypes observed in sh5 cells were due to ALCAM-silencing and not to off-target effects of the shRNA, we established the sh5rxd “rescue” line. The sh5rxd cells were transduced with retroviral constructs encoding both sh5 and a full-length, C-terminally GFP-tagged human ALCAM construct containing three silent point mutations, rendering it resistant to sh5 knockdown. Expression of the GFP-tagged ALCAM was validated by western blot using antibodies against either ALCAM or GFP, which confirmed that sh5rxd expressed the higher molecular weight GFP-tagged ALCAM but little, if any, endogenous ALCAM (Fig. 2B). As expected sh5rxd cells exhibited GFP- and ALCAM-positive cell-cell junctions (Fig. 2I–K and data not shown).

### ALCAM-silenced Cells Display Reduced Motility and Invasive Capacity

**Figure 3:**
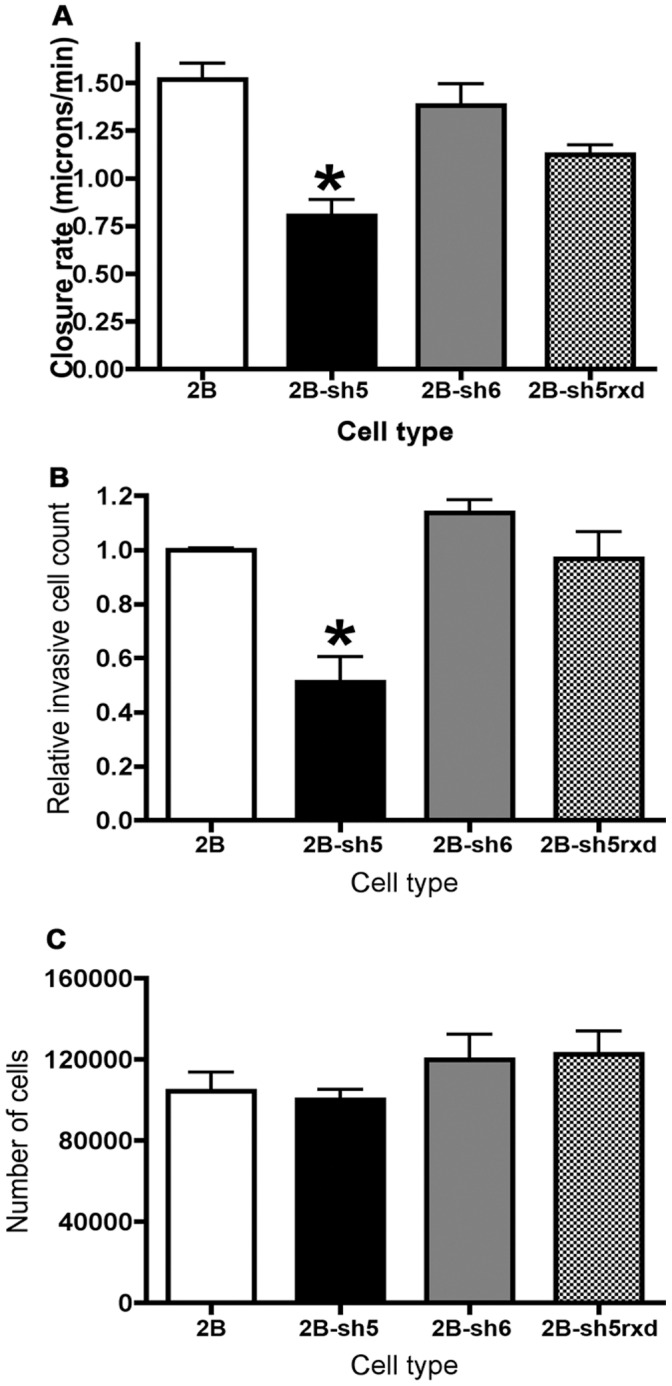
ALCAM-silenced cells display reduced wound-gap closure speed, invasive capacity, and MMP-2 activation. Closure rate of cells in a wound-gap assay was determined for each cell line listed (minimum of 3 trials each; A). The speed of sh5 cells was significantly reduced compared to MUM-2B, sh6 control, and sh5rxd rescue cells (ANOVA; p<0.05). Invasive capacity of each cell line was assayed via transwell migration. The number of invasive cells was standardized relative to MUM-2B for each cell line shown (minimum of 3 trials each; B). The average number of invasive MUM-2B cells per three non-overlapping 10× fields was 806. The sh5 cell line displayed significantly reduced invasive capacity compared to MUM-2B and sh5rxd rescue (ANOVA; p<0.01). To ensure that differences in invasiveness were not due to differences in growth or survival after plating, an equivalent number of cells was plated for each line. Eight hours later, the number of cells was assayed, and found to be comparable between lines (C). Error bars are mean ± S.E.M.

We first tested sh5 ALCAM-silenced cells in the gap closure assay previously described, comparing them to both native MUM-2B cells and sh6 control cells. Silencing ALCAM results in a significant reduction in motility: sh5 cells exhibit a closure rate nearly 50% lower than that of parental MUM-2B or non-silenced sh6 cells (Fig. 3A). Although their velocity was markedly reduced, the sh5 cells still appeared to move as a cohesive sheet, and individual cells did not detach from the invasion front as in MUM-2C (data not shown). We next sought to determine how silencing ALCAM impacts invasive capacity of MUM-2B uveal melanoma cells. To accomplish this, we used a commercial transwell assay (CytoSelect, Cell Biolabs, Inc.) comprising an upper chamber separated from a lower chamber by a basement membrane matrix-coated 8 µm (pore size) filter. A defined number of cells were placed in the upper chamber and the cultures incubated for 8 hours, following which the number of cells that had invaded the matrix and reached the underside of the filter was counted. As expected, the sh6 and sh5rxd cell lines did not exhibit any statistically significant difference in invasive capacity compared to MUM-2B (Fig. 3B). In contrast, ALCAM-silenced sh5 cells showed a 50% reduction in invasive capacity, consistent with the similar magnitude reduction in motility observed in the gap closure assay (Fig. 3B).

Because the invasion assays were performed over a period of 8 hours, it was formally possible that sh5 cells simply proliferated more slowly than MUM-2B, which might contribute to the difference in the number of cells counted on the underside of the transwell filter. To ascertain that this was not the case, we performed a cell survival assay by plating a known number of cells in standard tissue culture wells, incubating for 8 hours, and then counting the cells. No significant differences were found in the survival of MUM-2B, sh5, and sh6 cell lines after 8 hours (Fig. 3C) or in growth at 24 hours (data not shown). Thus, our experiments demonstrate that ALCAM expression is necessary for cell motility and invasiveness in MUM-2B uveal melanoma cells.

### ALCAM Overexpression is not Sufficient to Enhance Migration and Invasive Capacity in MUM-2C Cells

**Figure 4:**
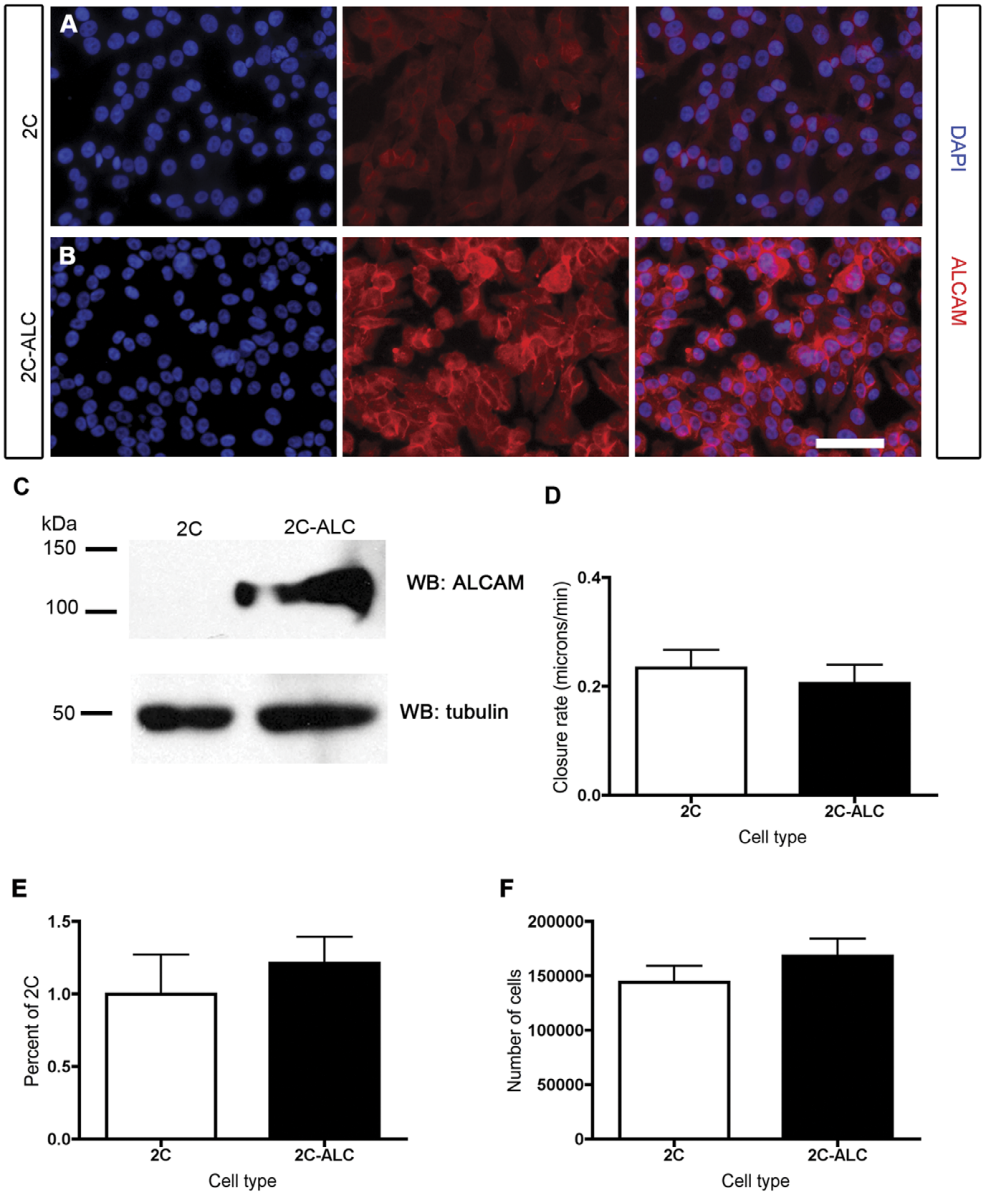
Expression of ALCAM in MUM-2C cells does not enhance wound-gap closure speed or invasive capacity. Immunostaining of MUM-2C cells (A) reveals that ALCAM expression is virtually undetectable in these cells. DAPI-stained nuclei are shown in blue; ALCAM staining is shown in red. In the 2C-ALC cell line (B), engineered to stably overexpress ALCAM, DAPI-stained nuclei are shown in blue, and ALCAM staining is shown in red. ALCAM localizes to points of contact between cells in the 2C-ALC cell line. The expression of ALCAM in 2C-ALC is confirmed by western blot in panel (C), and is undetectable in MUM-2C. Tubulin is shown as a loading control. The expression of ALCAM in 2C-ALC did not alter closure rate in a wound-gap assay (D), nor did it enhance invasive capacity of 2C-ALC cells when compared with MUM-2C in a transwell migration assay (t-test; p>0.05; the average number of invasive MUM-2C cells per three 10× fields was 16; E). Growth and survival of MUM-2C and 2C-ALC is similar, as assayed and described in Fig. 3 (F).

If ALCAM expression is necessary for motility and invasiveness in the MUM-2B uveal melanoma cell line, is ALCAM expression *sufficient* to increase motility and confer invasiveness in the normally ALCAM-negative MUM-2C line? To test this, we created a stable cell line, termed 2C-ALC, by transducing MUM-2C with a virus encoding full-length ALCAM. Expression of the full-length ALCAM construct was confirmed by both western blot (Fig. 4C) and immunohistochemistry (Fig. 4A, B). Expression level of ALCAM in 2C-ALC was roughly comparable to that of MUM-2B. As expected, ALCAM localized to cell-cell contacts in 2C-ALC cells (Fig. 4B). Overexpression of ALCAM in the 2C-ALC cell line, however, failed to enhance the velocity of cells in the gap closure assay (Fig. 4D). 2C-ALC cells still often moved as individual cells (similar to native 2C cells; Fig. 1F), and not as a cohesive sheet like MUM-2B cells (data not shown). Overexpression of ALCAM was also not sufficient to enhance the invasive capacity of 2C-ALC cells (Fig. 4E), nor did it affect the survival or proliferation of the cell line (Fig. 4F).

### ALCAM-silenced Cells Exhibit Reduced MMP-2

One likely way in which ALCAM could promote an invasive phenotype is through regulation of matrix metalloproteinases (MMPs). MMPs are zinc-dependent proteinases whose expression has been implicated in processes such as tissue remodeling and cancer metastasis. MMP-2, a 72 kDa protein also called gelatinase A, is the most abundant of the MMPs and is documented as a marker of poor prognosis in a variety of cancers [46]–[48]. Activation of MMP-2, and the additional gelatinase family protein MMP-9, allows degradation of type IV collagen basement membranes. MMPs are synthesized as pro-enzymes that must be processed to their active form by proteolytic cleavage. Pro-MMP-2 is recruited from the extracellular milieu and processed by a complex consisting of Type I MMP (MT1-MMP/MMP-14) and tissue inhibitor of metalloproteinase-2 (TIMP-2); this process is known to require full-length ALCAM [40]. Thus, we assayed MMP-2 levels in our panel of melanoma cell lines via gelatin zymography and western blot, where pro-MMP-2 appears as a 72 kDa band, and active MMP-2 appears as a ∼64 kDa band.

**Figure 5:**
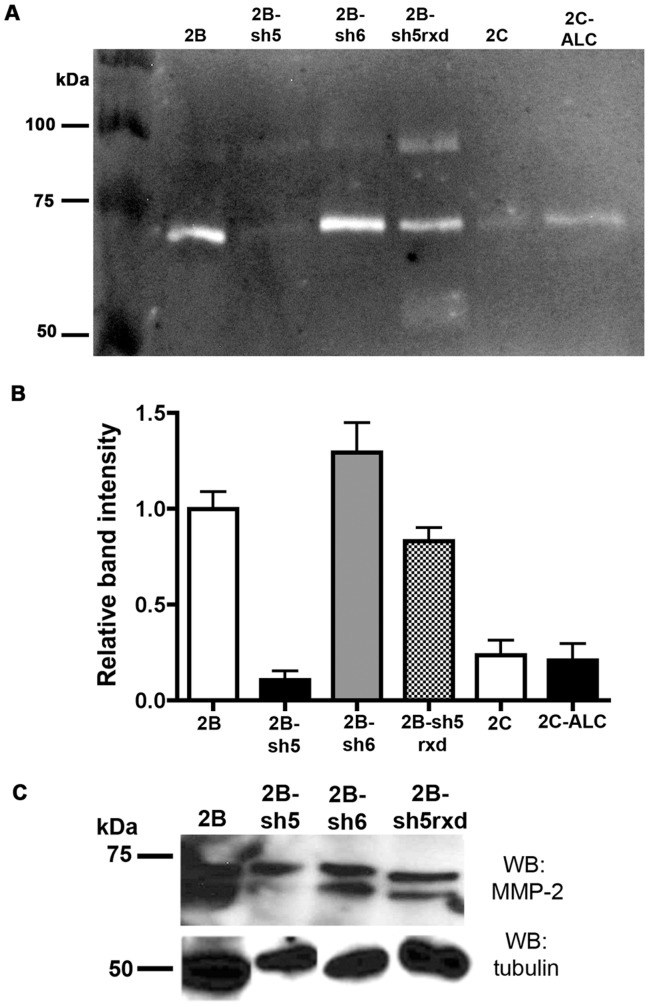
ALCAM-silenced cells display reduced MMP-2 activity. Levels of pro-MMP-2 were assayed in media conditioned by each cell line by gelatin zymography (A). Clear bands indicating pro-MMP-2 activity (which is activated by SDS; a faint “active” cleaved MMP2 band was present in some gels but often too weak for robust quantification) are present in MUM-2B, sh6, and the sh5rxd cell lines, but are reduced in the ALCAM-silenced sh5 cell line. MUM-2C and 2C-ALC display pro-MMP-2 levels that are lower than MUM-2B, and comparable to sh5 cells; the overexpression of ALCAM in 2C-ALC fails to increase pro-MMP-2 activity beyond that of MUM-2C. A minimum of three independent gelatin zymography trials (except 2C-ALC, 2 trials) are quantified in (B). (C) Western blots of cell lysates shows that the activation of pro-MMP-2 in the sh5 cell line is reduced (higher molecular weight band is pro-MMP-2; lower molecular weight band is active MMP-2) compared to MUM-2B, sh6, and the sh5rxd rescue cell line.

In gelatin zymography, active MMP-2 appears as a clear, Coomassie-negative band of ∼64 kDa upon staining of the gel; the “pro-MMP2” band of 72 kDa is also active in this assay, in the presence of SDS [40]. Because the pro-MMP2 band was much more prominent in our conditioned media samples, we quantified this band as a measure of MMP2 levels secreted by cells; in many gels, we could see a faint ∼64 kDa active band as well, which tracked levels of the clearer pro-MMP2 band (data not shown). Gelatin-clearing MMP-2 activity was strong in MUM-2B (Fig. 5A), C918, and M619 cells (data not shown), all of which highly express ALCAM (Fig. 1C), but not in the ALCAM-negative OCM-1A (data not shown) or MUM-2C (Fig. 5A). Next, we quantified MMP-2 activity in the stable cell lines, sh5, sh6, and 2C-ALC (Fig. 5A, B). We found that MMP-2 activation was reduced in sh5 by nearly 80% compared to parental MUM-2B, control sh6 cells, and sh5rxd rescued cells (Fig. 5B). As expected from our previous results with the 2C-ALC cell line (Fig. 4) MMP-2 activity was not increased in 2C-ALC compared to parental MUM-2C (Fig. 5A, B); again, this suggests that ALCAM is necessary, but not sufficient, for an invasive cell phenotype in uveal melanoma. Pro-MMP-2 was detectable in MUM-2B, sh5, sh6, and sh5rxd cell lysates by western blot, indicating that even sh5 expressed this enzyme (Fig. 5C). Consistent with the decreased invasive capacity in sh5, the active form of MMP-2 was just barely detectable in sh5, yet was clearly present in MUM-2B, sh6, and sh5rxd (Fig. 5C). It is possible that sh5 ALCAM-silenced cells exhibit defects in both MMP2 secretion and MMP2 activation, based on our combined results.

### Cadherin-based Junctions are Disorganized in ALCAM-silenced Cells

Another way in which ALCAM could influence tumor cell behavior is through the regulation of other adhesion molecules, particularly those that have been implicated in metastasis, such as classical cadherins (for reviews, see refs. [49]–[51]). Several previous lines of evidence support such a hypothesis. First, a report by Tomita and colleagues [52] described the coordinate recruitment of epithelial (E)-cadherin and ALCAM to cell contacts upon transfection of alpha-catenin into prostate cancer cell lines that have lost this protein. Second, ALCAM has been shown to colocalize with both alpha-catenin and filamentous actin in MV3-Tiam1 cells [53]. Finally, ALCAM could be co-immunoprecipitated with VE-cadherin and N-cadherin in PVMEC cells [54], and has been reported to be present in the same lipid microdomain compartments as cadherins [55].

**Figure 6:**
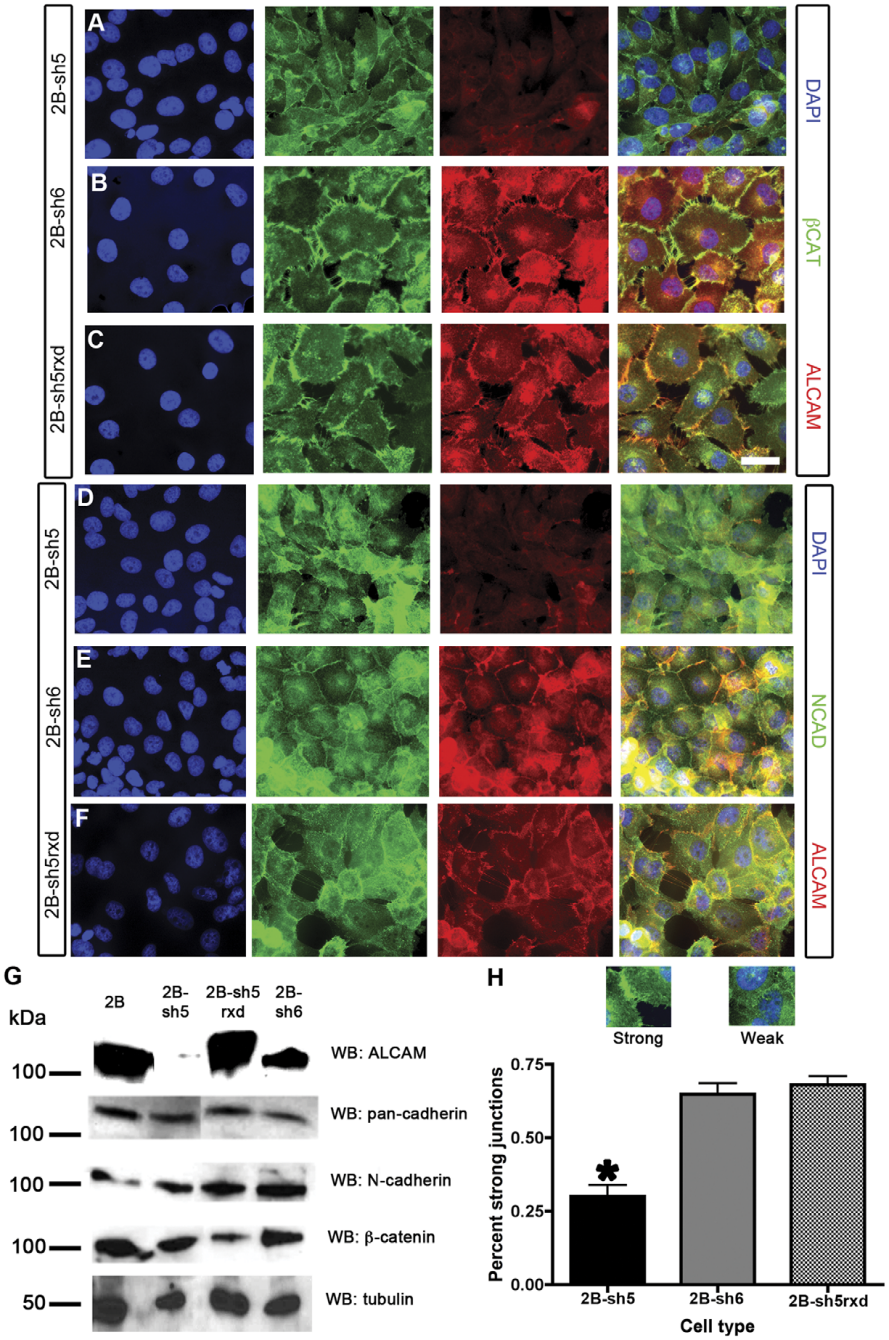
Silencing of ALCAM disrupts N-cadherin and ß-catenin junctions. (A-F) Immunostaining of sh5 (A, D), sh6 (B,E), and sh5rxd (C,F) cells for nuclei (DAPI; blue), ß-catenin or N-Cadherin (green), and ALCAM (red) reveal that adherens junctions are disorganized when ALCAM is absent (A, D). Control sh6 cells and sh5rxd rescue cells, in contrast, have strong localization of ß-catenin and N-Cadherin at cell-cell contacts (B,E; C, F), where ALCAM colocalizes. Overall levels of adhesion molecules were assayed in MUM-2B, sh5, sh5rxd, and sh6 by western blot (G). The levels of expression detected by pan-cadherin, N-cadherin, and ß-catenin antibodies were similar across all cell lines. The proportion of cells that exhibited strong ß-catenin-labeled cell-cell junctions was quantified and is shown in (H), as are representative examples of cell-cell junctions classified as strong or weak. The number of sh5 cells forming strong ß-catenin junctions is significantly reduced in sh5 compared to sh5rxd (t-test, p<0.0001). Error bars are mean ± S.E.M. Scale bars are 10 microns.

**Figure 7:**
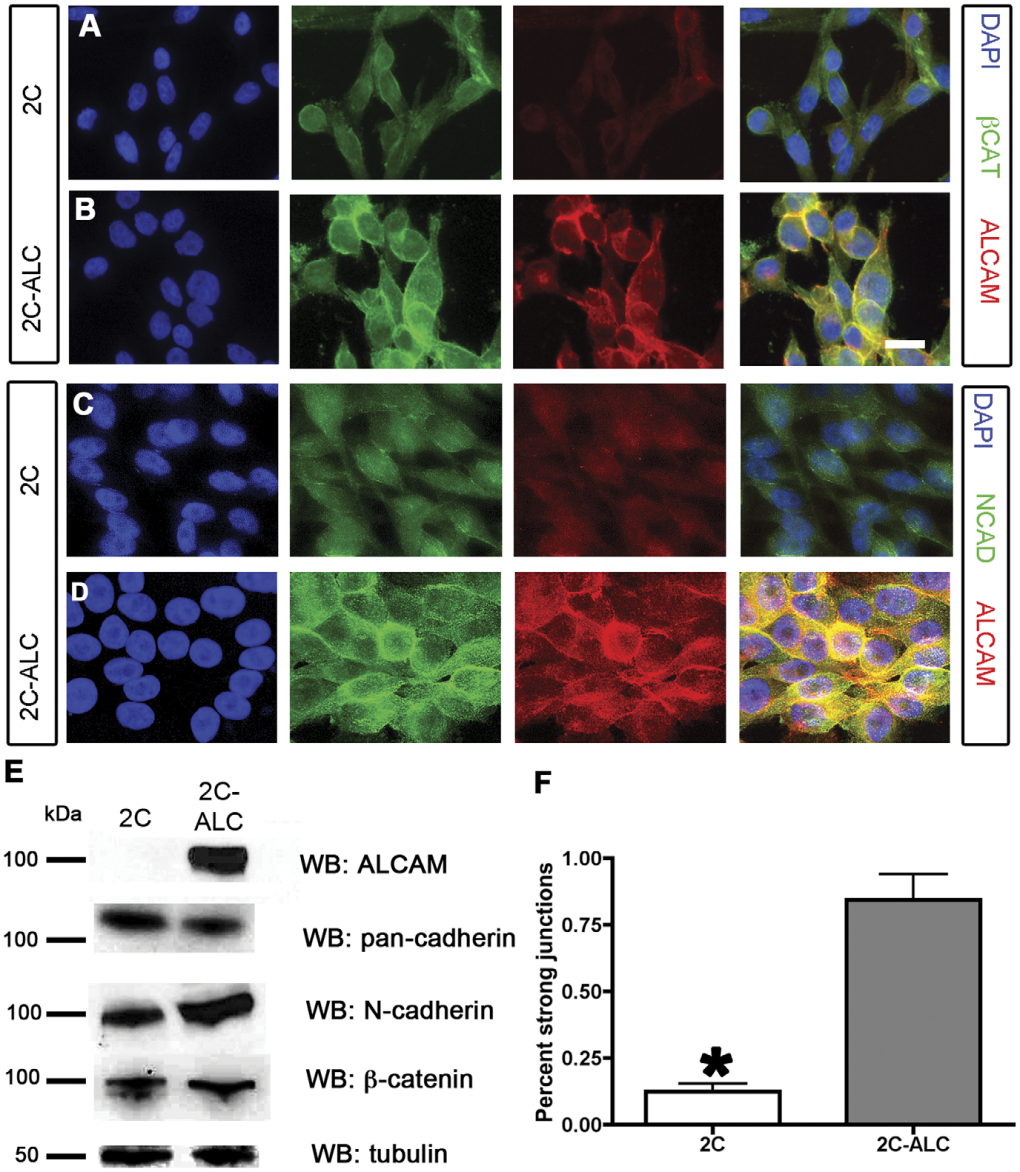
ALCAM expression in 2C-ALC cells enhances formation of N-cadherin and ß-catenin junctions. (A, C) Immunostaining of 2C cells for nuclei (DAPI; blue), ß-catenin or N-Cadherin (green), and ALCAM (red) reveal that ß-catenin is diffusely localized and not prominent at cell-cell contacts. In contrast, 2C-ALC cells (B,D) display enhanced localization of ß-catenin and N-Cadherin to cell-cell contacts in the presence of ALCAM expression. In the merged images, ALCAM colocalizes at adherens junctions and at points of contact between adjacent cells. Levels of adhesion molecules were assayed in MUM-2C and 2C-ALC by western blot (E). The levels of pan-cadherin, N-cadherin, and ß-catenin are similar in both cell lines. The proportion of cells forming strong ß-catenin positive junctions is shown in F. 2C-ALC cells have significantly more ß-catenin positive cell junctions than do MUM-2C cells (t-test; p<0.05). Error bars are mean ± S.E.M. Scale bars are 10 microns.

We began by assessing the expression of cadherins in uveal melanoma cell lines. Both MUM-2B (Fig. 6G) and MUM-2C (Fig. 7E) expressed N-cadherin as well as ß-catenin; neither had detectable levels of E-cadherin (data not shown). To determine whether ALCAM silencing affects adherens junctions, we compared N-cadherin and ß-catenin staining in sh5 (ALCAM-silenced), sh6 (control), and sh5rxd (ALCAM-silenced+rescued) cells. In parental MUM-2B cells (data not shown) or in sh6 control cells, strong ß-catenin (Fig. 6B) and N-cadherin (Fig. 6E) staining was localized to cell-cell contacts that were also ALCAM-positive, and cells had a flattened, epithelioid shape. In contrast, sh5 silenced cells had disorganized ß-catenin (Fig. 6A) and N-cadherin (Fig. 6D) junctions, a more spindle-like shape, and often grew on top of each other. This phenotype was significantly rescued by re-expression of ALCAM in the sh5rxd cell line (Fig. 6C, F). Quantification (examples of “strong” and “weak” junctions are shown in Fig. 6H) showed that the percentage of cells with contiguous, strong ß-catenin-positive adherens junctions was reduced from nearly 75% in control lines to ∼25% in sh5 (Fig. 6H). Neither silencing of ALCAM in sh5 nor its re-expression in sh5rxd appeared to affect levels of ß-catenin or N-cadherin expression (Fig. 6G).

This phenomenon was not restricted to uveal melanoma cells. Transient transfection of HEK293 cells with an shRNA construct (sh2) confirmed to silence ALCAM also resulted in reduced ß-catenin localization to cell-cell contacts, and a lack of strong junctions (Fig. S1). In HEK293 cells transfected with a negative control, scrambled shRNA (sh0), ß-catenin localization to cell junctions was not perturbed (Fig. S1).

### ALCAM Expression Enhances Cadherin-mediated Cell-cell Contacts in the 2C-ALC Cell Line

Finally, we addressed whether ALCAM expression would be sufficient to enhance the formation of cadherin-based adherens junctions in 2C-ALC cells. Parental MUM-2C cells exhibited weak, diffuse ß-catenin and N-cadherin staining, with few clear cell-cell junctions (Fig. 7A, C). Expression of ALCAM in the 2C-ALC cell line led to an increase in ß-catenin and N-cadherin staining along the length of cell junctions, which colocalized precisely with ALCAM (Fig. 7B, D). Despite this, cell morphology remained fairly similar to MUM-2C’s spindle shape, and no increase in total N-cadherin or ß-catenin levels was observed (Fig. 7E). In parental MUM-2C cells, only ∼15% of cells had ß-catenin-positive junctions; in contrast, over 75% of 2C-ALC cells did (Fig. 7F). Together with our analysis of sh5 silenced cells, these data suggest that ALCAM expression is both necessary and sufficient to promote the recruitment of N-cadherin and ß-catenin to form adherens junctions in uveal melanoma cells.

## Discussion

ALCAM has demonstrated functions in many critical developmental processes such as hematopoiesis [6]–[8], neurite outgrowth [13]–[21], retinal ganglion cell targeting [23], and T-cell activation [10]–[12]. ALCAM has also been implicated in pathological states, such as cancer metastasis, but its role remains somewhat confusing. ALCAM has been identified as a marker of metastasis in many tumor cell types, and yet in other cases it has been associated with inhibition of metastasis. Reports from the literature thus present a paradox regarding ALCAM’s relationship to tumor cell motility and invasiveness.

For example, initial studies described a positive correlation between ALCAM expression and metastatic capacity or progression of cutaneous melanoma [25], [32]. The role of ALCAM in cutaneous melanoma was first addressed directly by the laboratory of Guido Swart [56]. An amino terminal-truncated (dominant negative) form of ALCAM was transfected into cutaneous melanoma cells, and was found to diminish cell clustering and enhance both motility *in vitro* and the transition from primary tumor to tissue invasion *in vivo*. It appeared that the disruption of homophilic ALCAM contacts thus resulted in increased metastatic potential in cutaneous melanoma cell lines [56]; this was, however, in contrast to previous expression data that predicted ALCAM would *promote* invasion and metastasis.

When other cancer types are considered, the picture becomes murkier – one study by Kristiansen and colleagues [35] found that ALCAM protein expression is high in low-grade prostate cancer, and is lost in higher-grade tumors. A study of colorectal cancer demonstrated overexpression of ALCAM neoplastic regions compared to normal surrounding tissue; membranous ALCAM staining correlated with reduced patient survival [31]. Studies of breast carcinoma also provide seemingly conflicting results: one study [28] found that low ALCAM expression correlated with high tumor grade and metastasis, while another [29] showed that ALCAM is associated with smaller tumor diameter and grade.

Studies describing ALCAM function in uveal melanoma, the most common form of primary intraocular cancer, are lacking. Our aim in initiating this study was to determine the role that ALCAM plays in modulating invasiveness and motility in uveal melanoma, and to provide mechanistic data that will contribute to an understanding of why ALCAM up- and down-regulation might be associated with different stages of different cancers. We describe a correlation between ALCAM expression and motility in a gap closure assay in uveal melanoma cells. These data suggested that ALCAM plays a role in promoting motility and migration. We further find that silencing of ALCAM in the invasive MUM-2B line results in decreased motility, invasiveness, and MMP-2 activation.

Our results have implicated ALCAM as a regulator of cadherin-based adherens junctions in uveal melanoma cells. The disruption of N-cadherin/ß-catenin-positive junctions we observe in ALCAM-silenced sh5 cells is striking. Typically, N-cadherin and ß-catenin localize strongly to cell-cell contacts, colocalizing with ALCAM. In ALCAM-silenced cells, however, both N-cadherin and ß-catenin localization at cell contacts is markedly reduced – it appears as if adherens junctions “fall apart” in the absence of ALCAM. This is consistent with earlier findings by Ofori-Acquah and colleagues [54], in which ALCAM co-immunoprecipitated in multiple adherens junction complexes. We were not, however, able to co-immunoprecipitate ALCAM with N-cadherin in uveal melanoma cells, suggesting that any interaction may not be direct or may be sensitive to our lysis conditions.

The cadherins have long been implicated in invasion and metastasis, with N-cadherin/E-cadherin expression often dictating invasive potential in cancer cells. In addition to mediating intercellular and cell-matrix adhesive interactions, cell adhesion molecules also modulate signaling pathways. Thus, changes in the expression and localization of cell adhesion molecules can influence tumor progression by both modulating the adhesion status of a cell and by altering cell signaling. In many human cancer types, including melanoma, the loss of E-cadherin function is concomitant with expression of mesenchymal cadherins, including N-cadherin [57], [58].

N-cadherin has been shown to promote cell motility and migration – in stark contrast to the anti-migratory properties of E-cadherin [59], [60]. N-cadherin is capable of overcoming E-cadherin-mediated cell adhesion, resulting in induction of an invasive phenotype [59], [61]. This so-called ‘cadherin switch’ not only occurs during the transition of cancer cells to an invasive phenotype, but is also a hallmark of the epithelial-to-mesenchymal transition that occurs during embryonic development. Given that ALCAM expression can modulate N-cadherin localization at cell-cell junctions, we can envision the following possibilities as to how ALCAM status might influence the migratory and invasive properties of uveal melanoma cell lines.

ALCAM-induced N-cadherin junction formation might enhance the ability of tumor cells to move into different surroundings. Stromal cells, fibroblasts, and blood vessel endothelial cells express N-cadherin [62]. Positive regulation of N-cadherin junctions by ALCAM could allow cancerous cells to successfully move through adjacent N-cadherin-positive tissues by promoting interactions with these cells, thereby enhancing the probability of metastasis. This is an attractive hypothesis, given that the choroid of the eye is rich in blood vessels, and that endothelial cells express N-cadherin and vascular endothelial (VE)-cadherin [62]. When accompanied by a loss of E-cadherin, the tumor cells may lose their ability to interact with adjacent epithelial cell types. Therefore, expression of ALCAM could promote a state of dynamic adhesion, whereby cells dissociate from their primary site and subsequently interact with adjacent stromal cells and endothelial cells. In cases where adjacent cells are devoid of N-cadherin expression, we speculate that ALCAM expression might not serve to promote metastasis, as it would not increase the interaction between the two cell types. Since our cells were devoid of detectable E-cadherin expression, we were not able to observe whether ALCAM has a similar effect on this or other cadherin family members, though the literature suggests it does [52], [54].

In addition to modulating adhesion specificity, ALCAM-induced N-cadherin junction formation might also provide cells with a pro-migratory signal. N-cadherin is capable of inducing motility and invasion independent of E-cadherin status [61]. Breast cancer cells transfected with N-cadherin show increased metastatic potential when injected into nude mice [59]. What N-cadherin signaling pathway might lead to increased motility and invasion? Fibroblast growth factor receptors (FGFRs) have been shown to physically interact with N-cadherin, likely through interactions between the fourth extracellular domain of N-cadherin and the first two Ig-like domains of FGFRs [63], [64]. It is hypothesized that N-cadherin interaction with FGFRs facilitates binding of fibroblast growth factor-2 (FGF2) to its receptor but helps prevent internalization of FGFR. This, in turn, leads to increased expression of FGFRs at the cell surface, which contributes to sustained MAPK signaling. The end result is increased motility, invasiveness, and secretion of matrix metalloproteinases, including MMP-9 [64].

The cutaneous-derived BLM cell line, being devoid of cadherin expression, might not be subject to the same changes in signaling induced by decreasing ALCAM-ALCAM interactions as would our uveal melanoma cell lines. The reduction of homophilic ALCAM interactions in a cell line lacking cadherins might free the cells from interacting with each other, allowing migration of individual cells into surrounding tissue. The reduction in invasiveness we observe appears at odds with the finding that amino-truncated ALCAM expression served to disrupt ALCAM junctions and to reduce MMP-2 activation, but actually *increased* the invasive capacity of BLM cutaneous melanoma cells [56] [40].

An attractive hypothesis that could account for the increased invasiveness caused by a dominant-negative ALCAM [56] versus our own results in which silencing ALCAM results in decreased invasiveness, centers around the cadherin status of the cell lines used in each study. BLM cells are devoid of N-, E-, and P-cadherin expression [65], while both cell lines used in our study strongly express N-cadherin (but not E-cadherin; P-cadherin was not assayed).

Overall, our work confirms a previously suggested link between ALCAM and cadherins [52], [54], and provides a new example of the regulation of cadherins by IgSF members. Nectins are IgSF molecules that localize to adherens junctions in epithelial cells [66], [67], and influence E-cadherin-mediated adhesion [68], [69] appears to increase the overall strength of adhesion between cells. All nectins associate with an intracellular binding partner, afadin, which directly links nectins to the actin cytoskeleton [70]. Afadin also associates with alpha-catenin [71], [72]. As ALCAM’s intracellular interaction partners are completely unknown, a key component of our work going forward will be focused on identifying such partners, and the signaling pathways associated with them. It will also be important to determine the specificity of the interaction between ALCAM and cadherins: can silencing of ALCAM remove a variety of classical cadherins from adherens junctions in different cell types? Several reports have demonstrated that ALCAM and cadherins are present in the same lipid microdomains [5], [54], [55]: does ALCAM regulate cadherin recruitment to these rafts? New studies focused on identification of both extracellular and intracellular binding partners of ALCAM will be critical to understanding of the mechanisms by which ALCAM regulates adherens junctions, cell motility, and invasive capacity.

## Supporting Information

Figure S1 **Silencing of ALCAM in HEK cells results in disrupted ß-catenin junctions.** HEK cells were transiently transfected with an shRNA construct confirmed to silence ALCAM (sh2), or a negative control scrambled shRNA (sh0). Both constructs included a GFP marker to track transfected cells (pseudocolored blue). HEK cells with silenced ALCAM expression show reduced ß-catenin localization (green) to cell-cell contacts, as well as reduced ALCAM expression (red; asterisk indicates an untransfected cell with a higher expression level of ALCAM). HEK cells transfected with the negative control shRNA, however, display robust ALCAM expression that localizes to cell junctions, and ß-catenin localizes strongly to cell junctions in these cells.
(DOC)
