# Supplementary material for: ALCAM Regulates Motility, Invasiveness, and Adherens Junction Formation in Uveal Melanoma Cells
Source: PLoS One. 2012 Jun 26;7(6):e39330. doi: 10.1371/journal.pone.0039330 (PMC3383762; doi:10.1371/journal.pone.0039330)
Supplement: Figure S1 — Silencing of ALCAM in HEK cells results in disrupted ß-catenin junctions. HEK cells were transiently transfected with an shRNA construct confirmed to silence ALCAM (sh2), or a negative control scrambled shRNA (sh0). Both constructs included a GFP marker to track transfected cells (pseudocolored blue). HEK cells with silenced ALCAM expression show reduced ß-catenin localization (green) to cell-cell contacts, as well as reduced ALCAM expression (red; asterisk indicates an untransfected cell with a higher expression level of ALCAM). HEK cells transfected with the negative control shRNA, however, display robust ALCAM expression that localizes to cell junctions, and ß-catenin localizes strongly to cell junctions in these cells. (DOC) [file pone.0039330.s001.doc]

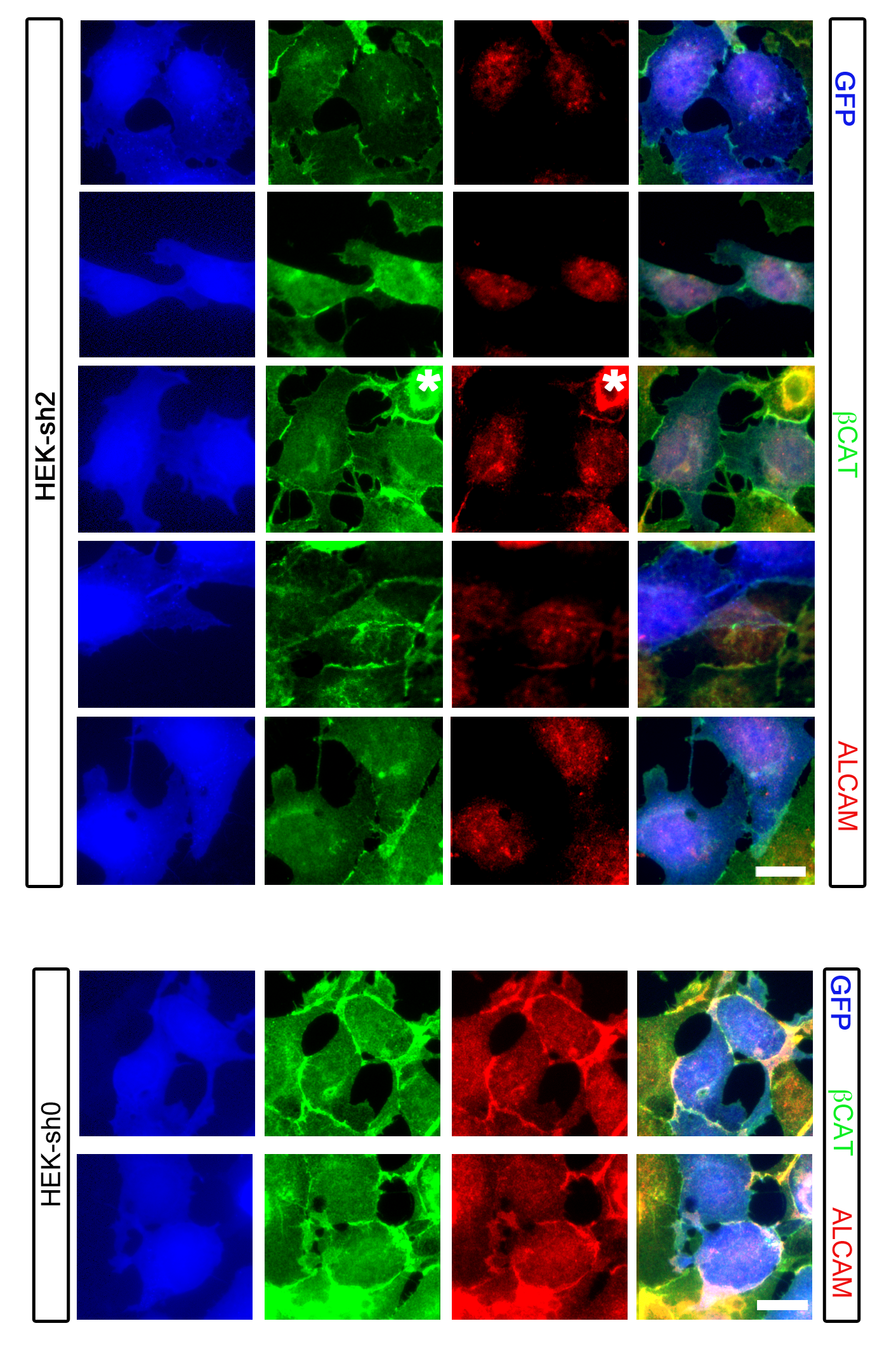
**Supplemental Figure S1: Silencing of ALCAM in HEK cells results in disrupted β-catenin junctions.** HEK cells were transiently transfected with an shRNA construct confirmed to silence ALCAM (sh2), or a negative control scrambled shRNA (sh0). Both constructs included a GFP marker to track transfected cells (pseudocolored blue). HEK cells with silenced ALCAM expression show reduced ß-catenin localization (green) to cell-cell contacts, as well as reduced ALCAM expression (red; asterisk indicates an untransfected cell with a higher expression level of ALCAM). HEK cells transfected with the negative control shRNA, however, display robust ALCAM expression that localizes to cell junctions, and ß-catenin localizes strongly to cell junctions in these cells.
